# Excellent outcomes with Oxford Uni-compartmental knee arthroplasty in anteromedial osteoarthritis patients (≤60 years) at mid-term follow-up

**DOI:** 10.1186/s12891-021-04747-y

**Published:** 2021-10-08

**Authors:** Zhen Li, Zhenyue Chen, Jinqiang Wei, Xianzhong Zeng, He Sun, Zehui Li, Xuewei Cao

**Affiliations:** 1 The Second Clinical Medical College of Guangzhou University of Chinese Medicine, Guangzhou, 510405 Guangdong China; 2 The First Clinical Medical College of Guangzhou University of Chinese Medicine, Guangzhou, 510405 Guangdong China; 3 Department of Orthopaedic Surgery, Guangdong Provincial Hospital of Traditional Chinese Medicine, 111 Dade Road, Yuexiu District, Guangzhou, 510120 Guangdong China

**Keywords:** Oxford Uni-compartmental knee Arthroplasty, 60 years, Impant survivorship, Mid-term follow-up

## Abstract

**Background:**

The use of Oxford uni-compartmental knee arthroplasty (UKA) has rapidly increased worldwide,however,the relevance of younger patients for postoperative function after Oxford UKA remains unclear. The main purpose of our study is to clarify the effectivemess of Oxford UKA in the younger Chinese patients with anteromedial osteoarthritis (AMOA).

**Methods:**

We retrospectively enrolled 252 consecutive patients who underwent Oxford UKA for AMOA with a minimum follow-up of 5 years between March 2013 and December 2016. The patients were divided into the younger (≤60 years) and elderly (> 60 years) age groups. The demographic data and surgery variables were recorded and compared. Patient satisfaction grade, range of motion (ROM), Oxford knee score (OKS), Hospital for Special Surgery (HSS) score, Western Ontario and McMaster (WOMAC) Universities Osteoarthritis Index score and postoperative complications were recorded. The 5-year survival of the implants were also compared with TKA revision as the endpoint.

**Results:**

A total of 252 consecutive patients were recruited, including 96 aged 60 years or less and 156 aged over 60 years. The mean follow-up duration in the younger and elderly groups were 73.6 months (SD,standard deviation, 4.1) and 74.7 months (SD 6.2) respectively. Patient satisfaction rate was high in both groups (*P* = 0.805). Furthermore, no significant differences were observed in postoperative ROM(*P* = 0.299), OKS(*P* = 0.117), HSS(*P* = 0.357) and WOMAC scores(*P* = 0.151) between the younger and elderly groups (*P>*0.05). However, the incidence of joint stiffness (*P* = 0.033) and delayed wound dehiscence (*P* = 0.026) were significantly different between both groups. Five-year implant survival without revision were also similar in both groups (96.9% vs 97.4%, *P* = 0.871), and that for the entire cohort was 97.2% (95% CI 95.4–99.6).

**Conclusion:**

Oxford UKA for AMOA demonstrated favorable results in younger patients aged ≤60 years at a minimum 5-year follow-up in terms of patient satisfaction, functional outcomes, implant survival and postoperative complications. Therefore, younger patients might not be considered as an absolute contraindication to Oxford UKA.

## Introduction

Oxford uni-compartmental knee arthroplasty (UKA) is a minimally invasive surgery that replaces the surface of the knee joint, and is an ideal treatment for isolated uni-compartmental osteoarthritis (OA). It is increasingly replacing total knee arthroplasty (TKA) on account of less blood loss, shorter operation time, faster postoperative recovery, fewer complications, knee proprioception and bone stock preservation [1–3]. The indications for UKA have also been expanded in recent years [3–6], and the high survival rates of the implants have been demonstrated [7–10].

Based on their experience with fixed-bearing devices, Kozinn and Scott [11] proposed age greater than 60 years, weight less than 180 pounds (82 kg), high activity requirement, patellofemoral joint or lateral compartment degeneration, cartilage calcium deposition, preoperative flexion angle > 90°, flexion contracture < 5°, and angular knee deformity < 15° as the indications for Oxford UKA. On the other hand, Goodfellow et al. [12, 13] established the indications for Oxford UKA based on the anteromedial osteoarthritis (AMOA) and spontaneous osteonecrosis of the knee (SONK). The ideal indications for AMOA patients are medial compartment OA with bone-on-bone wear, full thickness cartilage in the lateral compartment, and functionally normal anterior cruciate ligament, posterior cruciate ligament and medial collateral ligament. However, studies increasingly show that Oxford UKA can also be performed for younger patients without these classic indications [14–19].

In Caucasian populations, younger patients have demonstrated excellent functional outcomes after Oxford UKA [14, 20]. However, the functional outcomes and implant survival are ambiguous for younger Asian patients with completely different lifestyles and physical activities [20–22]. The aim of this study was to assess the mid-term patient satisfaction, functional outcomes, implant survival and postoperative complications of Oxford UKA in Asian patients aged ≤60 years, and to determine the ideal indications for this procedure.

## Materials and methods

### Participants

A total of 252 consecutive patients with AMOA that underwent medial Oxford UKA surgery were retrospectively recruited between March 2013 and December 2016. Follow-up period refered to the time from the end of surgery to June 2021.All patients had been operated on by one surgical team. The patients were divided into the younger (≤60 years) and elderly (> 60 years) age groups. The inclusion criteria were as follows: (1) primary severe AMOA with at least K-L grade three, (2) functionally intact ACL(Anterior Cruciate Ligament) and MCL(Medial Collateral Ligment), (3) correctable varus deformity (varus < 15°), (4) flexion deformity < 15° and an active range of motion (at least 0–100°), (5) intact lateral compartment with no evidence of OA, and (6) minimum 5 years of follow-up and availability of complete follow-up data. Patients with (1) bone groove-like changes on the lateral side of the patellofemoral joint (Qutebridge grade IV), (2) prior surgery on the same knee, and (3) rheumatoid arthritis or fixed varus deformity were excluded. Obesity, age, high activity or patellofemoral joint degeneration are not considered absolute contraindications. This study was approved by the institutional review committee of our hospital and informed consent was also obtained from all patients.

### Surgical procedure and perioperative management

Primary Oxford UKAs were performed using the Oxford Uni-compartmental Phase III mobile-bearing prosthesis (Biomet Merck Ltd., South Wales, UK) by the same group of surgeons. After administering general or spinal anesthesia, the patient was laid in a supine position, and the right lower limb was routinely disinfected. A tourniquet was applied to the proximal thigh on the operative side. Pump up the tourniquet before cutting the skin,and loosen the tourniquet before suturing the surgical incison to stop bleeding. The operative time refers to the time from cutting the skin to closing the wound,including two parts,prosthesis and wound suture. It takes about half an hour to install the prosthesis and half hour to close the wound. A tourniquet is only needed when installing a prosthesis,so the tourniquet time was far shorter than the operative time.

The knee joint was flexed to 90°, and an incision was made from the medial edge of the patella to 3 cm below the joint line. The surgery was performed as per the specifications of the Oxford Microplasty instrumentation. All patients received an analgesia cocktail formulated in our hospital, along with postoperative intravenous medication, acupuncture analgesia etc. The patients were instructed to walk within 2–6 h post-operation, and were given routine anticoagulant and antibiotics the following day. Rehabilitation treatment was started on the first day after surgery with walker-assisted movement, and full weight-bearing exercises were started 3 days later.

### Outcome measures

Patients were instructed to go to the outpatient clinic two and 4 weeks after discharge, and then 3, 6, 12 months and once a year postoperatively. Patient records were reviewed and the following data was collected: age, sex, operation side, body mass index (BMI), American society of anesthesiologist(ASA)classification,osteoarthritis(OA) grade and follow-up duration. The preoperative and postoperative values were compared. In addition, the anesthesia type, hospital stay duration, blood loss volume, tourniquet time and operative time were also recorded. At the final follow-up, patient satisfaction was documented as disappointed, dissatisfied, satisfied or very satisfied. Preoperative and postoperative range of motion (ROM), the Oxford knee score (OKS), hospital for special surgery (HSS) knee score and Western Ontario and McMaster (WOMAC) University osteoarthritis index score were used to evaluate the function of the knee joint at final follow-up. Postoperative complications such as patellar ligament injury, deep vein thrombosis, joint stiffness, wound infection, delayed wound dehiscence, radiographic lucency and revision rate were recorded and compared. The details of knee revision for TKA and the 5-year survivorship of Oxford UKA implants were also compared.

Postoperative radiographs of both groups were reviewed to determine the differences in implants. In the follow-up period,we analyzed the x-ray of each patients to find signs of complications,such as prosthesis position、size and dislocation, pathological radiolucent lines or lateral compartment OA progression. It is worth noting that the anterior radiograph should directly face the tibial prothesis,and the lateral radiograph should directly face the femoral prosthesis. Only in this way can we correctly compare the postoperative complications between the two groups from the x-ray.

### Statistical analysis

All statistical analysis was performed using GraphPad Prism 7.0 (GraphPad Software, San Diego, USA) and SPSS 23.0 (SPSS Inc., Chicago, IL, USA). Continuous variables were reported as mean ± standard deviation (SD). The inter-group differences of variables conforming to normal distribution were compared by independent sample *t*-tests. Categorical variables (demographic data, anesthesia type, satisfaction grade and postoperative complications) were described using frequency distributions and percentages, and compared by Chi-square test or Fisher’s exact test. Log-rank test was used to compare the Kaplan-Meier survival curves with revision to total knee arthroplasty(TKA)as the end point. All tests were two-sided and *P*-value < 0.05 was considered statistically significant.

## Results

**Fig. 1:**
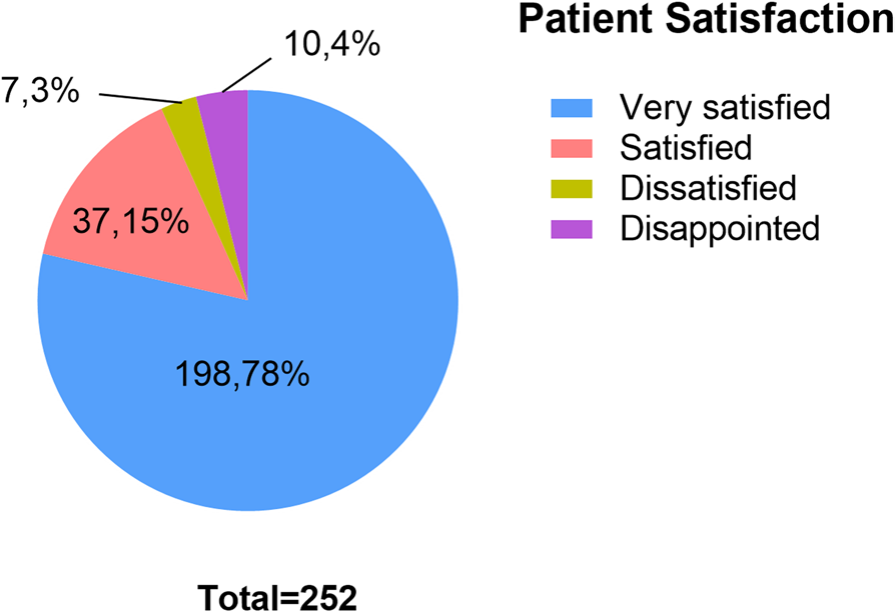
Patient satisfaction rate at a minimum 5-year follow-up after Oxford UKA

**Fig. 2:**
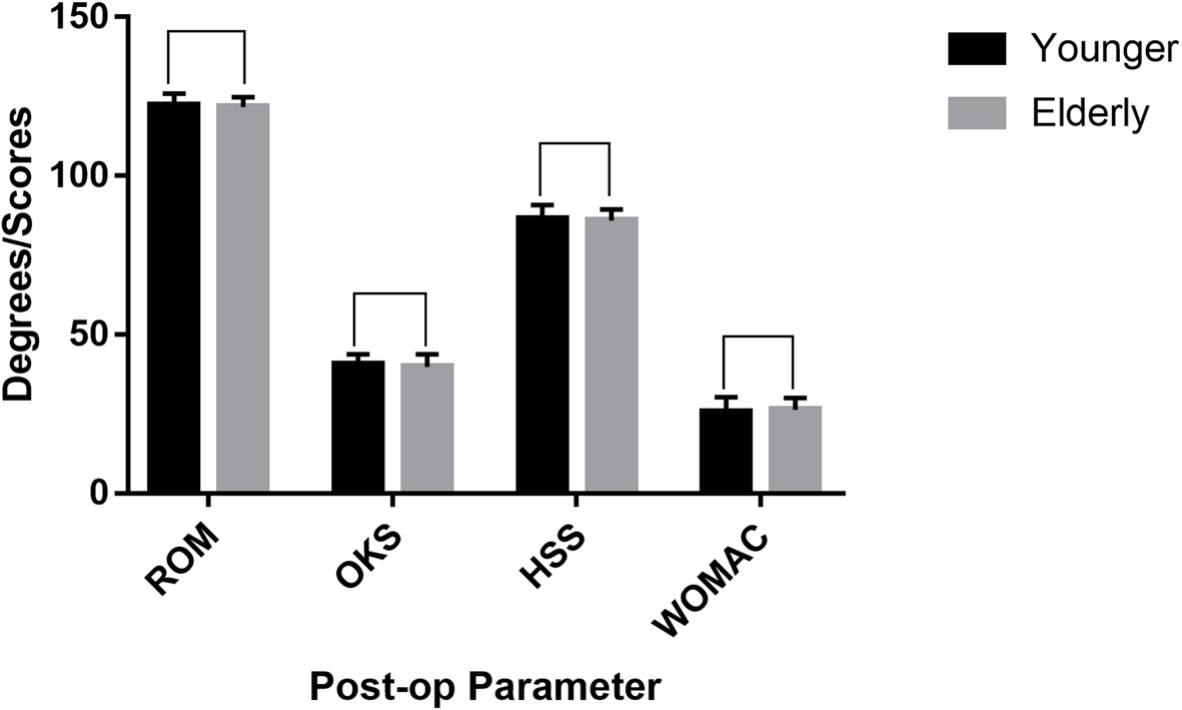
Functional outcomes of the two age groups after surgery measured in terms of ROM, OKS, HSS and WOMAC scores

Two hundred and fifty-two consecutive patients with a minimum 5-year follow-up were enrolled in this study, of which 96 were aged 60 years or younger, and 156 were over 60 years. The mean follow-up in the younger and elderly groups was 73.6 months (SD4.1) and 74.7 months (SD6.2) respectively. The demographic data of both groups are summarized in Table 1 and the clinical variables are listed in Table 2. There were no significant differences between the two age groups in terms of anesthesia type, hospital stay volume, blood loss volume, tourniquet time or operative time (*P* < 0.05). In addition, patients in both groups reported high satisfaction (*P* = 0.805), and the overall patient satisfaction rate was almost 93% (Fig. 1). Oxford UKA significantly improved the functional outcomes at follow-up compared to preoperative scores, and the postoperative ROM(*P* = 0.299), OKS(*P* = 0.117), HSS(*P* = 0.357) and WOMAC scores(*P* = 0.151) were similar in both groups (*P >* 0.05) (Fig. 2). For the elderly patients, the OKS, HSS and WOMAC scores improved by an average of 16.1, 26.3 and 19.8 points relative to preoperative values, and the mean improvement in these scores among younger patients were 16.3, 26.2 and 20.0 respectively (Table 3).

**Table 1:** Patients’demographic data between the two groups

Variables	Younger group(≤60 years)	Elderly group(>60 years)	*F* value/*x*^2^	*P* value
Knees	96	156	–	–
Age(years)	55.6 ± 4.0	69.0 ± 5.9	−19.70	< 0.001
Sex			0.231	0.631
female	68(70.8%)	106(67.9%)		
male	28(29.2%)	50(32.1%)		
Operation Side			0.632	0.427
right	56(58.3%)	83(53.2%)		
left	40(41.7%)	73(46.8%)		
BMI(Kg/m^2^)	26.9 ± 1.6	27.2 ± 2.7	−1.413	0.159
ASA classification			0.718	0.397
1 to 2	81(84.4%)	125(80.1%)		
3 to 4	15(15.6%)	31(19.9%)		
OA grade in MC(KL)			1.462	0.227
III	36(37.5%)	47(30.1%)		
IV	60(62.5%)	109(69.9%)		
Follow-up(months)	73.6 ± 4.1	74.7 ± 6.2	−1.521	0.130

Values are expressed as mean ± SD (standard deviation)or number (percentage); *BMI* body mass index, *ASA* American society of anesthesiologist, *OA* osteoarthritis, *MC* medial compartment, *KL* Kellgren-Lawrence score

**Table 2:** Comparisons of preoperative and postoperative variables between the two groups

Variables	Younger group(*n* = 96)	Elderly group(*n* = 156)	*F* value/*x*^2^	*P* value
Anaesthesia type			0.321	0.571
general	76(79.2%)	128(82.1%)		
spinal	20(20.8%)	28(17.9%)		
Hospital stay(d)	9.7 ± 1.9	10.2 ± 2.2	−1.73	0.086
Blood loss volume(ml)	82.6 ± 26.4	81.7 ± 23.3	0.258	0.796
Tourniquet time(min)	32.4 ± 3.0	33.1 ± 4.1	−1.569	0.118
Operative time(min)	82.0 ± 5.1	82.8 ± 4.7	−1.374	0.171

Values are expressed as mean ± SD or number (percentage)

**Table 3:** Comparisons of patient satisfaction and functional outcomes scores between the two groups

Parameter	Younger group (n = 96)	Elderly group (n = 156)	Mean difference	95% CI of mean diff	*F* value/*x*^2^	*P* value
Satisfaction grade					0.061	0.805
Satisfied-very satisfied	90(93.8%)	145(92.9%)				
Disappointed-dissatisfied	6(6.2%)	11(7.1%)				
ROM						
Preoperative	110.1 ± 3.4	109.2 ± 4.1	0.85	−0.12 to 1.83	1.720	0.087
Postoperative	122.2 ± 3.8	121.7 ± 3.1	0.48	−0.43 to 1.39	1.043	0.299
OKS						
Preoperative	24.3 ± 2.9	23.8 ± 3.7	0.44	−0.38 to 1.27	1.056	0.292
Postoperative	40.6 ± 3.3	39.9 ± 4.0	0.72	−0.18 to 1.62	1.571	0.117
HSS						
Preoperative	60.3 ± 3.7	59.7 ± 3.8	0.63	−0.33 to 1.58	1.296	0.196
Postoperative	86.5 ± 4.3	86.0 ± 3.5	0.48	−0.55 to 1.51	0.923	0.357
WOMAC						
Preoperative	45.5 ± 3.5	46.2 ± 4.3	−0.61	−1.64 to 0.42	−1.173	0.242
Postoperative	25.5 ± 4.8	26.4 ± 3.7	−0.83	−1.96 to 0.31	−1.443	0.151

Values are expressed as mean ± SD or number (percentage); *Diff* difference, *OKS* Oxford knee score, *HSS* hospital for surgery score, *ROM* range of motion

**Fig. 3:**
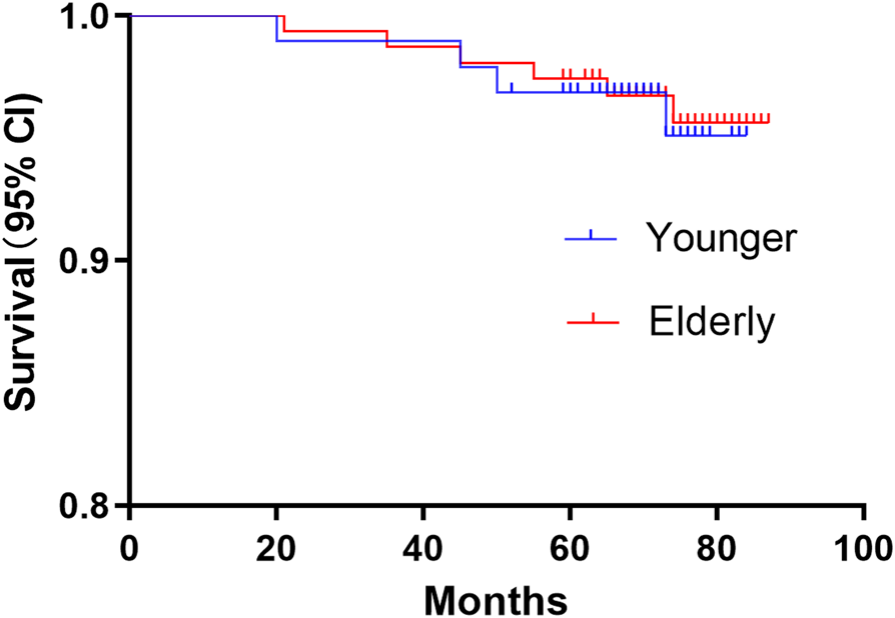
Kaplan-Meier survival curves with 95% confidence intervals in the younger and older patients with revision to TKA as the end point

**Fig. 4:**
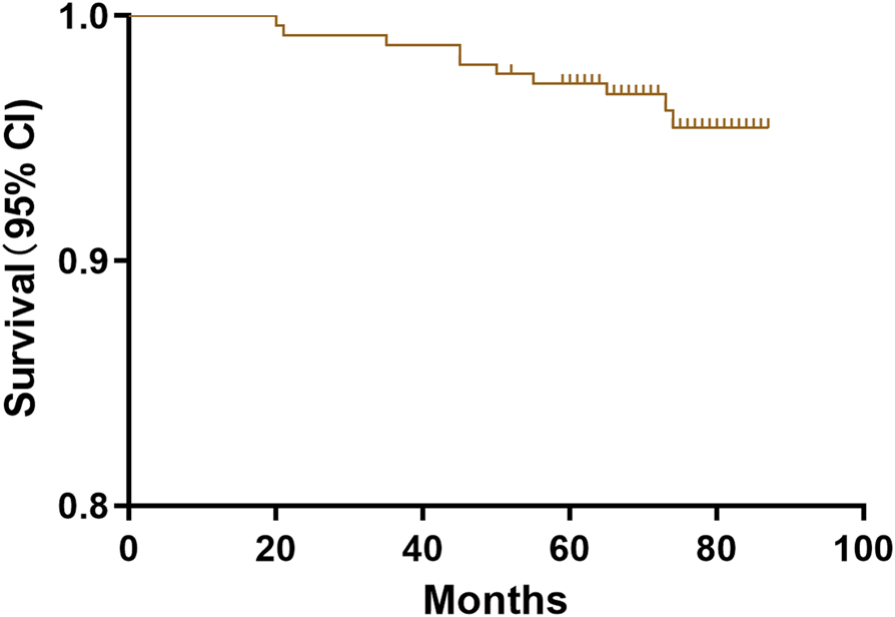
Kaplan-Meier survival curves 95% confidence intervals for all 252 consecutive patients with revision to TKA as the end point

**Fig. 5:**
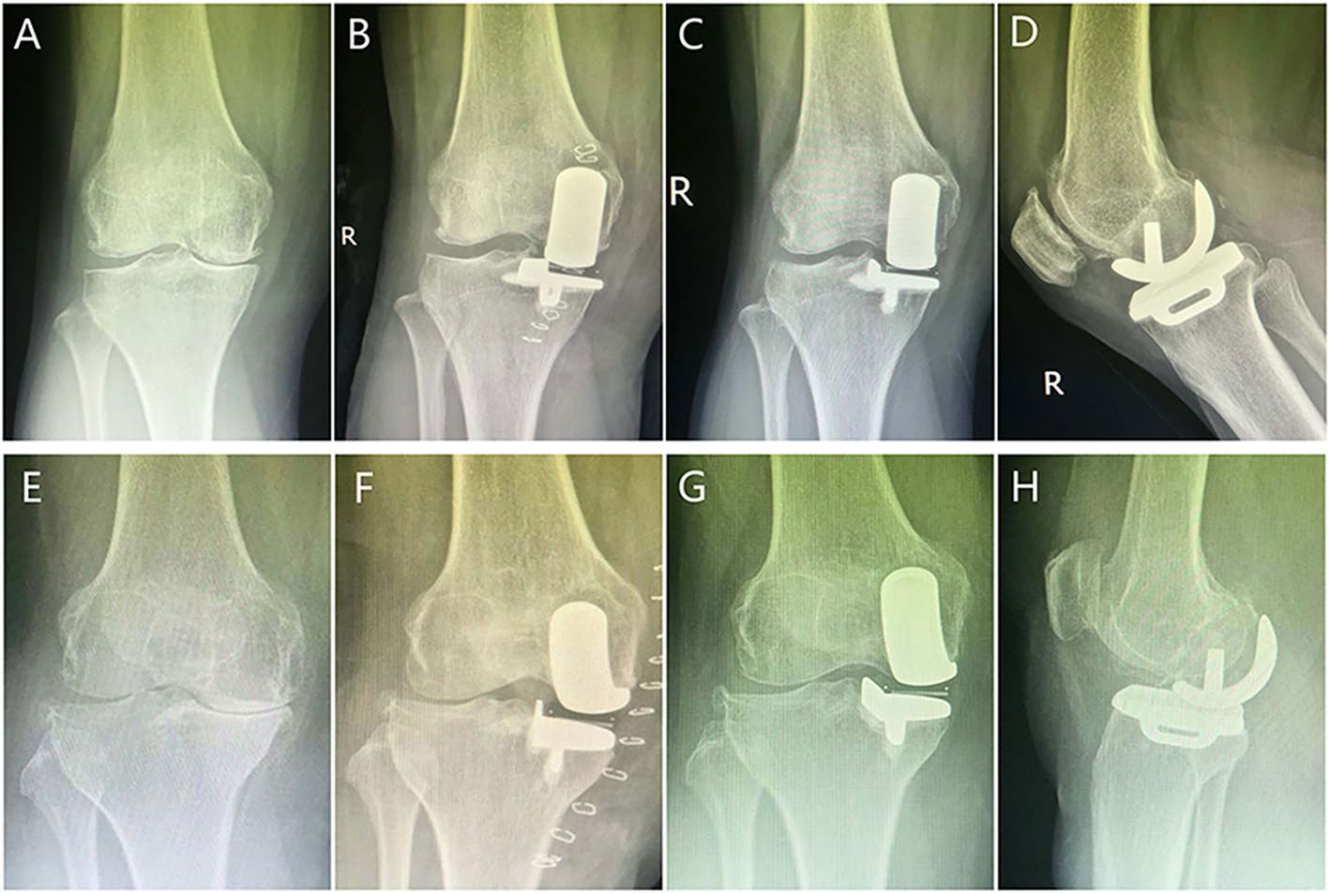
Radiographs of (A-D) a 54-year-old female patient and (E-H) a 69-year-old male patient with isolated compartment OA who underwent Oxford UKA and showed excellent outcomes at 5-year follow-up

There were no significant differences in the incidence rates of postoperative complications such as patellar ligament injury (1% vs 0%, *P* = 0.381), deep vein thrombosis(2.1% vs 1.9%, *P* = 1.000), wound infection (1% vs 1.3%, *P* = 1.000), radiographic lucency (4.2% vs 1.9%, *P* = 0.432) and revision rate (4.2% vs 3.8%, *P* = 1.000) between the younger and elderly groups (Table 4). However, the significant differences in terms of joint stiffness(*P* = 0.033) and delayed wound dehiscence (*P* = 0.026)were found in both groups (Table 4). As shown in Table 5, TKA was performed for 10 knees due to progression of lateral OA (5 cases, 50%), persistent unexplained pain (3 cases, 30%) or aseptic loosening (2 cases, 20%). Furthermore, seven knees required posterior-stabilized primary TKA implants, three knees required tibial stem and medial tibial augmentation was performed for one knee. Regardless of the reason, the mean time to TKA [47 (20–73) months vs 49.2 (21–74) months; (*P* = 0.878)] and 5-year survival rate of the implant [96.9% (95% CI 95.1–99) vs 97.4% (95% CI 95.6–99.4); *P* = 0.871] were similar in the younger and elderly age groups (Fig. 3). Furthermore, the 5-year implant survival rate was 97.2% (95% CI 95.4–99.6) for the entire cohort (Fig. 4). Comparison of the radiographs of a 54-years-old patient and 69-years-old patient indicated similar clinical outcomes at the 5-year follow-up (Fig. 5).

**Table 4:** Postoperative complications after Oxford UKA

Variables	Younger group(n = 96)	Elderly group(n = 156)	*P* value
Patellar ligament injury	1(1.0%)	0(0%)	0.381
Deep vein thrombosis	2(2.1%)	3(1.9%)	1.000
Joint stiffness	1(1.0%)	11(7.1%)	0.033
Wound infection	1(1.0%)	2(1.3%)	1.000
Delayed wound dehiscence	0(0%)	8(5.1%)	0.026
Radiographic lucency	4(4.2%)	3(1.9%)	0.432
Revision for any reason	4(4.2%)	6(3.8%)	1.000

Values are all expressed as number (percentage); *UKA* unicompartmental knee arthroplasty

**Table 5:** Details of the ten Knees Revised to TKA

Cases	Time to Revision(m)	age	Reason for Revision	TKA
1	20	58	Persistent unexplained pain	Primary PS TKA,no stems or augments
2	21	67	Persistent unexplained pain	Primary PS TKA,no stems or augments
3	35	75	Progression of lateral OA	Primary PS TKA,no stems or augments
4	45	59	Persistent unexplained pain	Primary PS TKA,no stems or augments
5	45	70	Progression of lateral OA	Primary PS TKA,no stems or augments
6	50	55	Aseptic loosening	PS TKA,tibial stem,no augments
7	55	67	Aseptic loosening	PS TKA,tibial stem,no augments
8	65	74	Progression of lateral OA	Primary PS TKA,no stems or augments
9	73	60	Progression of lateral OA	PS TKA,tibial stem and medial tibial augments
10	74	72	Progression of lateral OA	Primary PS TKA,no stems or augments

*OA* osteoarthritis, *PS* posterior stabilized, *TKA* total knee arthroplasty

## Discussion

The most significant finding of our study was that patient satisfaction, functional outcomes, implant survival and postoperative complications were similar in the younger and elderly OA patients at a minimum 5-year follow-up after Oxford UKA. Therefore,younger patients might not be considered as an absolute contraindication to Oxford UKA.

### Patient satisfaction

The overall patient satisfaction rate in our cohort was 93%, and was similar in both age groups. Several studies have reported high satisfaction in younger patients after UKA [15, 17]. Dalury et al. [16] reported satisfactory results for patients aged 46–59 years at mid-term follow-up. Mannan et al. [17] found that 97% of the patients aged 60 years or less were pleased or very pleased after UKA at minimum 10-year follow-up.

### Functional outcomes

Initial studies have shown that ideal candidates for UKA are older than 60 years of age [11]. However, we observed significant improvement and satisfactory functional outcomes in both age groups. Mannan et al. [17] reviewed a consecutive series of 91 medial UKAs performed in patients aged 60 years or younger at minimum 10-year follow-up, and demonstrated good-to-excellent long-term patient outcomes with 92.9 and 87.8% implant survival at 10 years and 15 years respectively. Lee et al. [18] also reported similar post-UKA outcome scores and implant survival in patients younger or older than 55 years at minimum 10 years follow-up. Hamilton et al. [23] evaluated 1000 Oxford UKAs and did not find any significant differences in the functional outcomes of patients aged younger and older than 60 years of age at 10-year follow-up. Walker et al. [19] reviewed 118 knees following medial mobile bearing UKA in patients aged 60 or younger and showed that the patients were able to resume normal physical activities, and nearly two-thirds could engage in high-level activities. Thompson et al. [5] reported that patients younger than 60 years appeared to have better results at 2-year follow-up, and Kleeblad et al. [24] showed that patients younger than 65 years were satisfied with the outcomes of UKA and TKA. Therefore, younger age is not a contraindication for UKA.

Given that squatting and sitting on the floor with high flexion angles are common daily activities in the Asian population, their post-operative functional requirements are greater compared to that of Western populations. Yoshida et al. [25] found that the 10-year implant survival rate in 1279 Japanese patients who underwent Oxford UKAs was 95%. Lim et al. [26] likewise reported a 94% 10-year implant survival rate in Korean patients. Xue et al. [27] assessed 708 consecutive medial Oxford UKAs in Chinese patients and found that the 5-year cumulative survival rate was 98.8% and the 10-year survival rate was 94.3%. The clinical outcomes of the patients in our cohort were similar to that of Western patients, and satisfactory ROM was achieved for daily activities.

### Implant survival

A recent review of twenty-six studies on 42,791 knees reported 95.3 and 91.3% 5-and 10-year pooled survival rates respectively after medial UKA [7]. Mohammad et al. [8] reported a 10-year survival of 93% and 15-year survival of 89% for 15 studies involving 8658 knees. Walker et al. [10] reported that medial Oxford UKA ensured a survival rate of 92.4% at 10 years and 88.6% at 15 years with an excellent functional outcome. Xue et al. [27] also reported 98 and 94.3% 5-and 10-year cumulative survival rates in Chinese patients. Several studies have also confirmed that patient age is not a determinant of implant survival, which is consistent with the 96.9 and 97.4% survival rates observed for the younger and elderly patients respectively at the 5-year follow-up. The Oxford mobile-bearing implant was used for all patients, indicating that it is a suitable choice for UKA.

The survival rate of UKA implant is usually evaluated with TKA revision as the endpoint. The major reasons for the failure of Oxford UKA are weight-bearing dislocation, aseptic loosening, lateral compartment osteoarthritis progression, and pain with no identifiable etiology. Xue et al. [27] reported that the most common reason of revision in Chinese patients were dislocation and osteoarthritis of the lateral compartment. Liddle et al. [28] reported that aseptic loosening was the most common reason for revision after UKA, and progression of arthritis and weight-bearing dislocation were almost exclusive to UKA. Other studies have implicated lateral compartment osteoarthritis progression and pain as the most frequent causes of revision [8, 9]. van der List et al. [29] found that aseptic loosening lead early failure of mobile-bearing UKA, whereas OA progression lead to the failure of fixed-bearing UKA in later years. Crawford et al. [6] also reported aseptic loosening as the most common early indication for revision of UKA, and arthritic progression as the most common indication in middle and late stages. In our study, progression of lateral OA, persistent unexplained pain and aseptic loosening were the most frequent causes of.

implant failure and revision.

### Ideal indications

The outcome of surgery and revision rate is directly determined by the surgeon’s experience and proficiency [30, 31]. In a review of 37,131 UKAs conducted in England and Wales, Liddle et al. [32] found that surgical caseload was a determining factor of implant survival, and the revision rate between surgeons with the lowest and highest-caseload had a four-fold difference. Kozinn and Scott [11] recommended strict inclusion criteria for UKA, whereas Goodfellow et al. [12, 13] recommended Oxford UKA for AMOA and SONK. In fact, with the increasing range of applications and excellent functional outcomes, the traditional indications of UKA have been expanded in recent years [3–6]. Thompson et al. [5] optimized the indications proposed by Kozinn and Scott based on the findings that younger patients had better clinical outcomes, and obese patients had a low revision rate at short or medium-term follow-up. In a large case series, Hamilton et al. [23] reported more indications based on the occurrence and development stage of the disease as proposed by Goodfellow et al. [12] instead of the contraindications set forth by Kozinn and Scott.

The aim of this study was to discuss the mid-term patient satisfaction, functional outcomes, implant survival and postoperative complications between younger and elderly groups. On the one hand,there were no obvious differences between the two groups in terms of patient satisfaction, functional outcomes and implant survival,which indicated that the indications for UKA have been expanded. There are many reasons for the expansion of UKA’s traditional indications. Among them,the surgeon’s experience and proficiency are crucial,and the development of prostheses and devices also play an important role. With the advancement of surgical technology,the surgical outcomes will be better,and the complications will be less than before. On the other hand,there were obvious differences in terms of postoperative complications including joint stiffness and delayed wound dehiscence,which may be related to the different age attributes of the two groups. The younger age group is presumed to have higher physical activity and the elderly age group is presumed to have low immunity.

## Conclusion

Oxford UKA for AMOA demonstrated favorable results in younger patients aged ≤60 years at a minimum 5-year follow-up. Patient satisfaction, functional outcomes, implant survivorship or postoperative complications were largely similar in both age groups. Therefore,younger patients might not be considered as an absolute contraindication to Oxford UKA.

### Limitations

The sample size of the study was relatively small, and the younger patients accounted for only 38% of the total cases. Secondly, the relative mid-term follow-up could not fully explain the exact difference between the two groups. Thirdly,we did not consider that different age groups have different attributes,such as physical activity and immunity,which may bias the study results. Further studies on larger cohorts with samples randomization and long-term follow-up are required to improve the difference between age groups.

## Data Availability

The datasets analyzed during the current study are available from the corresponding author on reasonable request.

## Abbreviations

UKA: Uni-compartmental knee arthroplasty
AMOA: Anteromedial osteoarthritis
SONK: Spontaneous osteonecrosis of the knee
ACL: Anterior Cruciate Ligament
MCL: Medial Collateral Ligment
ROM: Range of motion
OKS: Oxford knee score
HSS: Hospital for Special Surgery score
WOMAC: Western Ontario and McMaster Universities Osteoarthritis Index score
SD: Standard deviation
BMI: Body mass index
ASA: American society of anesthesiologist
OA: Osteoarthritis
MC: Medial compartment
KL: Kellgren-Lawrence score
PS: Posterior stabilized
TKA: Total knee arthroplasty
